# Impact of Rectus Diastasis Repair on Abdominal Strength and Function: A Systematic Review

**DOI:** 10.7759/cureus.12358

**Published:** 2020-12-29

**Authors:** Jessica Gormley, Andrea Copeland, Haley Augustine, Charlotte Axelrod, Mark McRae

**Affiliations:** 1 Plastic Surgery, Michael G. DeGroote School of Medicine, Hamilton, CAN; 2 Plastic Surgery, McMaster University, Hamilton, CAN; 3 Plastic Surgery, University of Toronto Faculty of Medicine, Toronto, CAN

**Keywords:** rectus diastasis, plication, strength, function

## Abstract

Rectus diastasis plication performed during abdominoplasty aims to narrow the widened linea alba and return the rectus muscle bellies to their anatomic position. It is unclear whether plication improves abdominal strength and function.

This systematic review summarizes the effect of rectus plication on abdominal strength, function, and postoperative complications.

A comprehensive search of CINAHL, Embase, Medline and Web of Science was performed. Screening and data extraction were performed in duplicate. Data were extracted from the included articles, and outcomes were analyzed categorically.

A total of 497 patients from seven articles were included. Mean age was 44.5 years (range 20.5-72) and 94.4% were female. Three articles reported abdominal strength measurements, with two showing significant improvement. Four articles used the SF-36 survey, all demonstrating improvement in physical function subscale postoperatively. An additional six instruments were used to assess functional outcomes, of which four demonstrated significant improvement. The overall complication rate was 17.0%.

Rectus plication is commonly performed during abdominoplasty to improve abdominal form and function. While the literature to date is encouraging with respect to functional outcomes, improvements in abdominal strength are less consistent. Heterogeneity in patient population, outcome measures, and comparison groups limit the strength of our conclusions. Future research should include a large comparative study as well as a protocol for standardizing outcomes in this population.

## Introduction and background

Abdominal rectus diastasis (ARD) is the separation of the rectus muscles due to thinning along the linea alba [1]. This separation may be caused by abdominal obesity, pregnancy, or congenital collagen abnormalities, and can lead to a permanent increase in the width of the aponeuroses from the patient's baseline by several centimetres [2]. ARD results in biomechanical compromise in the integrity of the abdominal wall, which has both aesthetic and physical consequences [3]. The primary aesthetic complaint is midline bulging of the abdominal wall above and below the umbilicus [4]. ARD may also result in decreased core strength, which can impact physical function and quality of life [5,6]. Poor posture, low back pain, decreased lung function, and urinary incontinence have also been reported from ARD [2,7,8].

Plication of ARD is often performed in abdominoplasty procedures [9]. Complaints such as back pain, abdominal pain, abdominal weakness, and midline bulge are indications for ARD repair [10]. Techniques used for repair vary widely based on surgeon preference and can differ in the suture material used, number of layers of closure, use of tension sutures, and use of mesh reinforcement [3].

Whether ARD plication improves abdominal strength and functionality is controversial. Several studies have demonstrated improvement in patients’ disability related index (DRI), objective core strength, lower back pain, measured posture, and quality of life [2,11-14]. However, other reports demonstrate no significant difference in functional, psychological, or physical results using health-related quality of life measures [6]. Furthermore, even if abdominal strength and function improve, it may be temporary, as the recurrence rate of ARD has been reported as high as 40% [3].

Alternatives to surgical intervention for symptomatic ARD have also been investigated. A recent systematic review highlighted several exercise programs that yielded modest improvements in both diastasis width and functional outcomes. However, due to the poor quality of the current literature and heterogeneity in outcome reporting, no new recommendations were made in this review [15]. A three-armed randomized trial by Emanuelsson et al. compared two surgical techniques - double rowed Quill suture plication and retro-rectus polypropylene mesh - to patients undergoing a three-month physiotherapy training program. All three groups improved their baseline abdominal strength per the Biodex system-4, which entails measuring patient-applied force against the system at various positional angles [10]. However, the operative groups surpassed the physiotherapy group in visual analog scale (VAS) and patient-perceived strength and saw greater improvements in the Biodex-4 system ratings. There were no differences between the two surgical techniques in terms of subjective strength or functional outcomes [10].

The current literature regarding abdominal strength following ARD repair is inconclusive and to the best of our knowledge, there is no systematic review on the subject. The objective of this study is to determine whether ARD repair improves abdominal wall strength and function in patients undergoing abdominoplasty compared to the patient’s baseline or other interventions.

## Review

Methods

Protocol and Registration

This systematic review adheres to PRISMA guidelines [16] and was registered a priori to Open Science Framework (OSF, 10.17605/OSF.IO/H9JB3) and can be found in Appendix A.

Search Strategy

A comprehensive search of CINAHL, Embase, Medline and Web of Science was completed from database inception to April 22, 2020, and was checked regularly for new relevant articles, with the assistance of a health science librarian. Our sample search strategy can be found in Appendix B. The search was limited to English language and human studies. A manual search of the included study’s references was completed to ensure relevant articles were not missed.

Article Selection

The following inclusion criteria were applied: 1) primary research; 2) patients undergoing abdominoplasty with open ARD repair; 3) abdominal strength or functional outcomes were reported; and 4) mean follow-up was greater than six months. Articles were excluded if: 1) they were case reports, opinion pieces, editorials and non-primary research (e.g., systematic reviews, scoping reviews, commentaries); 2) no strength or functional outcomes were reported; 3) a laparoscopic technique was used for ARD repair; 4) full text was not available; and 5) they were not written in English. When outcomes of interest were incompletely reported, an attempt was made to contact the authors for this data.

Study Screening

Title and abstract and full-text screening were performed in duplicate by two independent reviewers. Discrepancies at the title/abstract stage resulted in automatic inclusion in the next stage, and discrepancies at full text were resolved by consensus between the reviewers. Further discordance at this stage was then settled by the senior author. Reasons for exclusion at both the title/abstract and full-text stage were recorded. At each stage, reviewer agreement was assessed by calculating Cohen’s Kappa (κ) statistic [17].

Data Extraction

Data were extracted independently by two reviewers into an online collaborative spreadsheet (Google, California, USA). Data extracted included study characteristics (design, date, location, sample size, demographics, time horizon, and level of evidence), description of the population, intervention, comparator (if applicable), relevant outcomes, and time horizon.

Statistical Analysis

Due to the nature of the outcomes reported in the included articles, the analysis and results are presented in a descriptive fashion. Outcomes were grouped into the following categories: strength (primary outcome), functional, and complications. Categorical data were reported using frequencies and percentages. Continuous data were reported with weighted means, median and range.

Risk of Bias

Risk of bias for randomized control trials was assessed using the CLARITY group Cochrane standardized risk of bias assessment for randomized controlled trials [18]. For non-randomized trials, the Methodological Index for Non-Randomized Studies (MINORS) was used, with a minimum score of zero, and a maximum score of 16 for non-comparative studies or 24 for comparative studies [19].

Results

Included Articles

**Figure 1:**
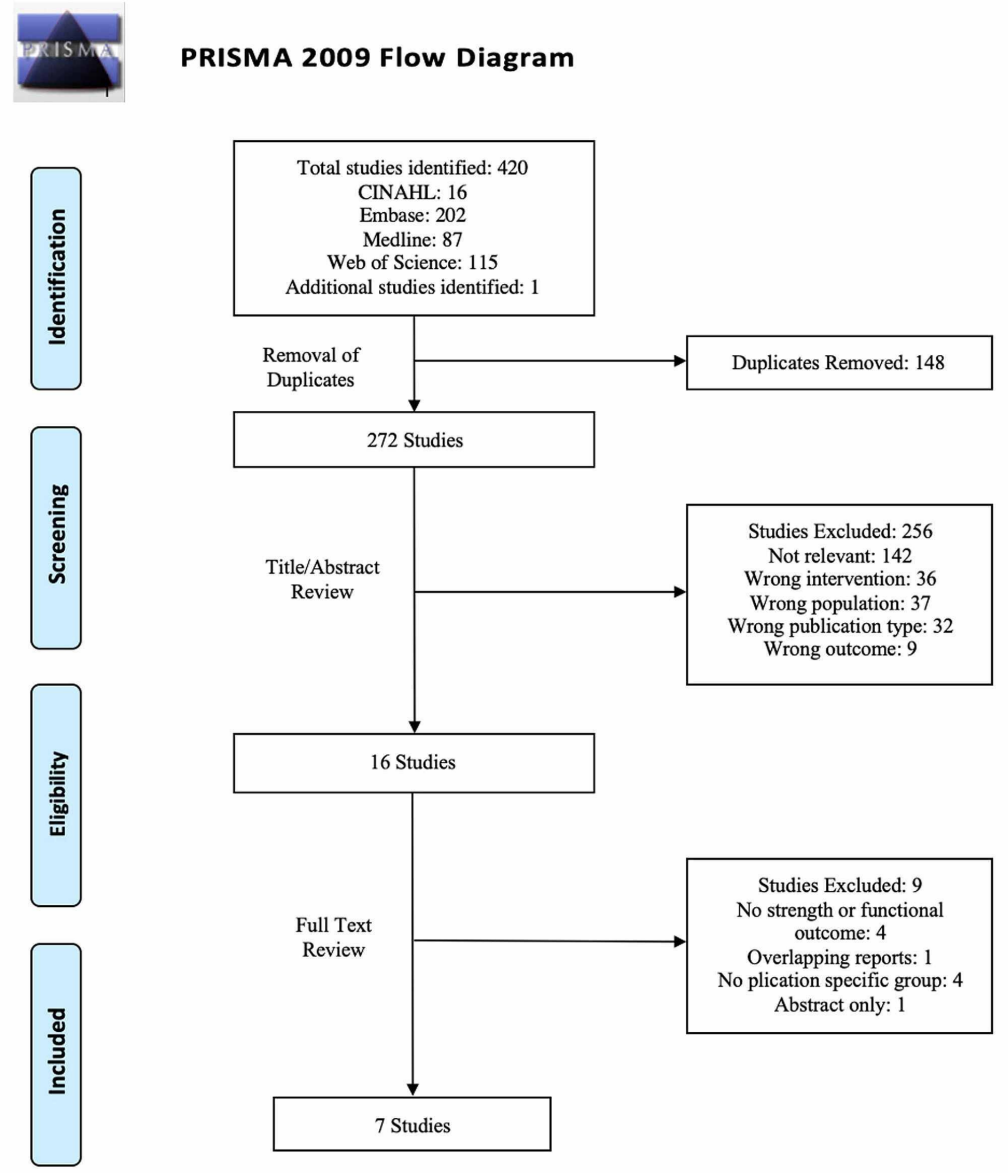
PRISMA Flow Diagram for Study Screening

The search yielded 420 articles, of which seven were included for analysis (Figure 1). Moderate agreement was achieved at the title/abstract stage (κ=0.761, 95% CI 0.590 to 0.932), and perfect agreement at the full-text stage (κ=1.00). Of the included articles, four were randomized control trials (Level I) [10], one was a retrospective cohort study (Level III) and two were prospective case series (Level IV). Two of the included randomized trials were overlapping reports of the same cohort of patients, with varying follow-up periods and outcome measurements [10,20,21]. The mean MINORS score for the two non-comparative articles was 9.5/16 (SD 0.71, range 9-10), which represents a moderate risk of bias. For the one non-randomized comparative study, the MINORS score was 15/24, which represents a moderate risk of bias. Study demographics can be found in Table 1, and PICOT summaries of included studies can be found in Table 2.

**Table 1:** Study Demographics *Median, **12-month follow-up for operative groups, three-month follow-up for training group. RCT: Randomized control trial.

Study	Study Design	Level of Evidence	Participants (N)	Mean age (range)	% Female	Mean Follow-up (months)
Emanuelsson et al. [10]	RCT	I	86	42.0* (27-66.9)	97.8	12 and 3**
Fiori et al. [22]	Retrospective cohort	III	94	41 (27-66)	97.9	12* (Range 4-24)
Olsson et al. [23]	Prospective case series	IV	56	38.8 (20.5-53)	100	12
Staalesen et al. [6]	RCT	I	96	43.3 (21.4-68.1)	83.3	12
Swedenhammar et al. [21]	RCT	I	52	42.5* (29-63)	96.2	60.2 (Range 45.6-78)
Temel et al. [7]	Prospective case series	IV	40	42.8 (33-48)	100	12 (Range 4-18)
Wilhelmsson et al. [2]	RCT	I	125	48 (25-72)	91.2	12

**Table 2:** PICOT Summaries ARD: Abdominal rectus diastasis; QoL: Quality of life.

	Population	Intervention	Control	Outcomes	Time Horizon
Emanuelsson et al. [10]	Patients with ARD and functional disability such as abdominal pain, weakness. For female patients, at least one previous pregnancy.	Group 1: retro-muscular mesh repair. Group 2: double-row Quill sutures	Group 3: training program (strength exercises) for three-month duration	Primary: ARD recurrence. Secondary: Abdominal muscle strength, pain, quality of life	1 year
Fiori et al. [22]	Patients with ARD >50 mm with or without umbilical hernia	Open repair (laparoabdominoplasty or laparominiabdominoplasty) with rectorectus mesh and suture closure of linea alba	Totally endoscopic sublay anterior approach (TESAR)	Primary: Quality of life (EuraHS-QoL)	1 year
Olsson et al. [23]	Postpartum women with diagnosed ARD and training-resistant symptoms	Double-row plication with Quill suture	Patient’s baseline	Primary: Abdominal trunk function. Secondary: Quality of life, ARD recurrence, urinary incontinence	1 year
Staalesen et al. [6]	Post-bariatric surgery patients	Abdominoplasty with ARD repair	Abdominoplasty with no ARD repair	Quality of life measures (SF-36, EuroQOL-5d)	1 year
Swedenhammar et al. [21]	Patients with diagnosed ARD combined with discomfort and/or abdominal pain	Group 1: retro-muscular mesh repair. Group 2: double-row Quill sutures	Patient’s baseline	Primary: long-term ARD recurrence. Secondary: Abdominal muscle strength, pain, and quality of life	5 years
Temel et al. [7]	Patients with excess abdominal skin, rectus diastasis, and substantial weakness of anterior abdominal wall	Abdominoplasty with ARD repair and liposuction	Patient’s baseline	Primary: postural outcomes. Secondary: back pain, quality of life, psychologic	1 year
Wilhelmsson et al. [2]	Patients with planned abdominoplasty due to excess skin	Abdominoplasty with ARD repair	Abdominoplasty with no ARD repair	Trunk endurance, self-rated physical function, and lung function	1 year

Patient Characteristics

There were 497 unique patients across all studies. The mean age was 44.5 years (SD 4.1, range 20.5-72) and 94.4% were female (469/497 patients). The median follow-up was 12 months (range 3-78 months), with one article having a mean follow-up less than 12 months. The pre-operative ARD width ranged from 3.0 to 10.0 cm. The mean preoperative body mass index (BMI) was 25.7 kg/m^2^ (SD 1.69, range 17.2-36).

Abdominal Strength Outcomes

Details of strength and functional outcome scales can be found in Table 3. Objective and subjective measurements of abdominal strength were reported in three studies, and the details of each study can be found in Table 4 [1,18,20]. Overall, rectus plication did not consistently improve abdominal strength. While Emanuelsson et al. [10] and Olsson et al. [23] demonstrated significant improvements in both objective (Biodex-4 system; abdominal endurance), and subjective (Visual Analogue Scale) strength measurements, Wilhelmsson et al. [2] found no significant difference in postoperative abdominal endurance between those who received plication and those who did not.

**Table 3:** Description of Outcome Measures

Study	Strength Outcomes	Functional Outcomes
Emanuelsson et al. [10]	Subjective improvement in abdominal strength measured with Visual Analogue Scale (VAS), where 0 represents no improvement and 10 represents maximal strength. Objective measure of abdominal strength using Biodex-System 4: patient secured with straps and seated at varying positional angles using anatomic landmarks, with the patient applying force against the machine with a pre-designed movement speed. Isokinetic measurement of force (Nm).	Quality of life was evaluated with the SF-36. The 8 domains addressed in SF-36 were physical functioning (PF); role limitations due to physical health (RP); bodily pain (BP); general health perceptions (GH); vitality (VT); social role functioning (SF); emotional role functioning (RE); and mental health (MH).
Fiori et al. [22]	None	Quality of life was evaluated with EuraHS-QoL. This is a hernia-specific questionnaire with nine questions scored by the patient from 0 to 10. There are three domains: “Pain” (range 0–30), “Restriction of activities” (range 0–40), and “cosmetic” (range 0–20). Total score ranges from 0-90, with a lower score being more favourable.
Olsson et al. [23]	Objective measure of abdominal endurance, conducted by physiotherapist using pre-validated stamina scale, measured in seconds.	SF-36 (as above). Physical functioning assessed using a self-rate disability related index (DRI) that included 12 non-specific activities of daily life. Each one is rated on a visual analog scale of 0 to 100, with 0 being no difficulty and 100 being inability to perform the task.
Staalesen et al. [6]	None	SF-36 (as above). Physical, functional and psychological assessed using the Modified Sahlgrenska Excess Skin Questionnaire, which assesses the impact excess skin has on patient’s experience and discomfort. Questionnaire was modified for this study to focus on excess abdominal skin rather than general.
Swedenhammar et al. [21]	None	SF-36 (as above).
Temel et al. [7]	None	Quality of life was assessed using the Nottingham Health Profile (NHP), which includes 38 questions on fatigue, pain, emotions, sleep, socialization and physical abilities.
Wilhelmsson et al. [2]	Objective measure of abdominal endurance: measured with the patient lying supine with knees bent at 90 degrees. Head and shoulders were raised until inferior border of scapula was off the table, and the number of seconds for which this position was held was recorded in seconds)	Disability Related Index (as described above).

**Table 4:** Study Outcomes NR: Not recorded; SF-36: Short Form (36) Health Survey; PF: Physical function; RP: Role-physical; BP: Bodily-pain; VT: Vitality; VAS: Visual analog scale; NHP: Nottingham health profile; Nm: Newton-meters.

Study	Strength Outcomes	Functional/Physical Outcomes
Emanuelsson et al. [10]	VAS: Median improvement in VAS: 7 (range 0-10) for sutured group, 8 (range 0-10) for mesh group, and 3 (range 0-10) for training group. Improvement in strength was significantly greater in operative groups compared to non-operative group (p < 0.001). No difference was seen between the two operative groups (p = 0.86). Biodex System-4: In all three groups, there was an improvement compared to pre-intervention values (p-values NR). The type of intervention did not affect magnitude of improvement (p for flexion 0.102, extension 0.149 and isokinetic 0.697).	SF-36: Pre-operatively, scores were significantly lower than Swedish norms (p < 0.001) in both operative groups. Post-operatively, scores reached Swedish norms. Similarly, scores were significantly lower than Swedish norms (p < 0.05) among the training group prior to the training intervention. Post-intervention, scores reached Swedish norms in all but one domain, bodily pain, in which they scored higher, indicating more pain (p-value NR).
Fiori et al. [22]	NR	EuraHS-QoL: There was a significant decrease in both groups in all post-operative values compared to the pre-operative scores at final follow-up, not only for the total score (p-value <0.01), but also for the pain, restriction and the cosmesis (p-values <0.01). No significant difference between the open or endoscopic groups was found at 6 or 12 months for total score (p-value 0.92), pain (p-value 0.51), restriction (0.43) or cosmesis (p-value 0.97). Open group: Total score pre-op 60.5 (16-84) to 1.0 (0-62) at 12 months (p-value <0.01) TESAR group: Total score pre-op 39.0 (21-72) to 2.0 (0-17) at 12 months (p-value <0.01)
Olsson et al. [23]	Endurance: There was a significant improvement in abdominal muscle strength [pre-operative: 49 seconds (range 0-240); post-operative 66 seconds (range 15-240); p < 0.001]	DRI: There was significant improvement (p < 0.001) in mean DRI score pre- vs. post-operatively. 98% of patients (55/56) reported fewer problems, with total scores on average being 79.1% lower at follow-up than before surgery. SF-36: Pre-operative scores were significantly lower than the general Swedish female population (p < 0.003), whereas postoperatively, scores were similar to Swedish female population in all subscales (p-values NR) except bodily pain, in which they scored higher indicating more pain (p < 0.001).
Staalesen et al. [6]	NR	SF-36: In the plicated group, there was significant improvement compared to pre-operative scores in one domain (physical function) (p = 0.022). However, there was no significant difference in improvement of this domain when compared to the non-plicated group or to Swedish norms. Modified Sahlgrenska Excess Skin Questionnaire: In the plicated group, there were significant improvements in all 10 domains. However, when compared to the non-plicated group, there were no significant differences in any domain. EuroQoL-5D: No significant changes in the EuroQol-5D dimensions or scores were observed in either the plicated or non-plicated groups.
Swedenhammar et al. [21]	NR	SF-36: Both operative groups showed significant improvement in all domains in long-term follow-up compared to pre-operative scores (p-values NR).
Temel et al. [7]	NR	NHP: There were significant improvements in fatigue, pain, and sleep (p < 0.001) but p-values were not calculated for the remaining categories including physical abilities.
Wilhelmsson et al. [2]	Endurance: Abdominal endurance showed no significant difference between pre- and post-operative abdominal strength in both the plication (p = 0.10) and non-plication groups (p = 0.17). There was no difference in pre- versus post-operative measurements for either the plicated or non-plicated group. Additionally, there was no difference between groups in post-operative measurements (p = 0.53). Non-plication pre: 58.9 (63.9) and post: 66.4 (69.8) Plication pre: 75.9 (70.1) and post: 89.7 (78.7)	DRI: There were no significant differences between pre- and post-operative scores in either the plication (p = 0.18) or non-plication (p = 0.38) groups. There was also no difference between plication and non-plication groups in physical function postoperatively (p = 0.35)

Functional Outcomes

Functional outcome measures were used in all seven studies; details of each study can be found in Table 4. The most consistent result was an improvement in the physical function subscale of SF-36 in all studies that used this instrument [6,10,21,23]. The next most commonly used scale, the DRI, showed improvement in one study [23] but no improvement in another [2]. The remaining functional scales (VAS, NHP, Modified Sahlgrenska Excess Skin Questionnaire, EuraHS-QoL) showed significant improvement after plication, except for the EuroQoL-5.

Complications

Post-operative complications were reported in five articles in patients who had received rectus plication (276 patients), with an overall complication rate of 17.0% (47/276). Of the 47 complications recorded, the three most common complications were seroma (12 patients, 27.7%), wound dehiscence (11 patients, 23.4%), and minor bleeding episodes (10 patients, 21.3%). An outline of the remainder of complications is found in Table 5.

**Table 5:** Postoperative Complications ARD: Abdominal rectus diastasis; NR: Not recorded.

Study	Complications
Emanuelsson et al. [10]	5 seromas (3 Quill, 2 mesh), 1 ARD recurrence (1 Quill), 26 (81.25%) dissatisfied with training only, and were offered operative repair. Rate: 11% (operative groups)
Fiori et al. [22]	16 total patients presented with one or more complications Open repair: 1 major bleeding, 10 minor bleeding, 2 umbilical necrosis, 1 pneumonia, 11 wound dehiscence TESAR: 1 seroma. Rate: 27.7%
Olsson et al. [23]	2 surgical site infections, 4 seromas, 4 hematomas, 1 pneumothorax. Rate: 19.6%
Staalesen et al. [6]	NR
Swedenhammar et al. [21]	No postoperative complications
Temel et al. [7]	2 surgical site infections, 2 seromas. Rate: 10%
Wilhelmsson et al. [2]	NR

Comparative Articles: Plication vs. No Plication Abdominoplasty

Two articles [2,6] compared patients who received rectus plication during their abdominoplasty to those who did not. Staalesen et al. found no significant differences between groups for the SF-36, the Modified Sahlgrenska Excess Skin Questionnaire, or the EuroQoL-5D (p > 0.05) [6]. Similarly, Wilhelmsson et al. [2] demonstrated no significant differences between the plicated and non-plicated groups in postoperative abdominal muscle endurance (p = 0.53), lung function (p = 0.25) or physical function (p = 0.35). The only difference observed was a significant improvement in postoperative running in the plicated group, which was not observed in the non-plicated group (pre-op DRI running score: 36, post-op: 8, p = 0.04) [2]. Unfortunately, neither article reported on complications and therefore we were unable to assess whether adding rectus plication affects the complication rate.

Discussion

The purpose of this study was to determine whether ARD improves abdominal wall strength and function in patients undergoing abdominoplasty. Overall, rectus plication did not consistently improve abdominal strength. In two of the three reporting studies [10,23], there was a significant improvement in both objective and subjective abdominal strength compared to the patient’s baseline. However, a third study by Wilhelmsson et al. demonstrated no significant difference [2]. Regarding functional outcomes, the most consistent result was an improvement in the physical function subscale of SF-36 in all studies that used this instrument [6,10,21,23]. The DRI scale showed improvement in one study [23] but no improvement in another [2]. Four of the remaining five functional scales demonstrated a significant improvement after plication, with the only exception being no significant improvement in EuroQoL-5 scores. With respect to postoperative complications, the overall complication rate in this review was 17.0%. This is consistent with current literature on postoperative complications following abdominoplasty ranging from 10 to 20% [24,25].

For abdominal wall defects, such as ventral hernias, it has been shown that restoring muscular continuity improves truncal strength and abdominal wall function [26,27]. Although a ventral hernia and a severe ARD, in theory, could have a similar impact on abdominal wall function, the biomechanics of ARD repair in abdominoplasty have not been studied. We postulated that ARD repair would enhance abdominal strength and function following abdominoplasty. However, the existing literature is of too poor quality and studies are too heterogeneous to make any strong conclusions. Additionally, the existing literature does not differentiate based on the severity of rectus diastasis, and therefore we are unable to determine the degree of defect that results in weakness, pain, or disability. It is possible that larger improvements could be expected in those with more severe diastasis, but this cannot be determined from the current literature. Furthermore, rectus plication could theoretically increase operative risk due to longer operative time and an increase in intraabdominal pressure; however, there were no data comparing complication rates between abdominoplasty alone versus abdominoplasty with plication, so this could not be assessed in this review.

The major limitation of this review is the heterogeneity and quality of outcome measures. Abdominal strength is measured in newton-metres (Biodex System-4), seconds (endurance), and subjectively with a visual analogue scale. This heterogeneity was not anticipated for our primary outcome, which was chosen a priori. The Biodex System-4 has proven to be a favourable outcome measure with high reliability and external validity [28]; however, only one article used this tool for assessment of abdominal strength [10]. Limitations of this method include cost and access to the equipment required [28]. The endurance testing uses a validated stamina scale but requires a trained physiotherapist and can be influenced by patient motivation [2,23]. Functional outcomes were measured using seven different scales assessing quality of life and physical functioning with a series of subjective patient-reported questionnaires. These tools are influenced by patient perception and are therefore limited in both their reliability and validity compared to objective measurements [29,30]. Only one of the seven scales (the Modified Sahlgrenska Excess Skin Questionnaire) was modified to be specific for the targeted patient population [6]. Another scale was specific for a similar patient population (the EuraHS-QoL for hernia patients) [22]. The most consistently reported functional outcome scale was the SF-36, which has been widely used in the scientific literature across a variety of patient populations [31]. However, the studies included in this review report a limited amount of SF-36 data, and therefore we were limited in the data available for pooled analysis [6,10,21,23]. To address this heterogeneity in outcome reporting, outcome standardization, which is being undertaken in several areas of plastic surgery, would be beneficial [32-35].

In addition to heterogeneity in outcome measures, there was substantial variation in the control groups used. Rectus plication was compared with no plication [2,6], mesh repair [10], endoscopic repair [22], and/or physiotherapy [10], and in three studies [7,21,23], there was no comparison group. This made it difficult to compare outcomes across studies even when the same outcome measure was used. Finally, there was variability in patient population (for example, post-partum women versus post bariatric surgery patients) [6,23]. Overall, heterogeneity precluded a meta-analysis.

One strength of our review is that there were three level I RCTs and two of the remaining three studies collected data prospectively. However, all three RCTs had high risk of bias in at least one domain, and two had high risk of bias in more than one domain. Of the non-randomized non-comparative trials [7,23], the mean MINORS score was 9.5/16, indicating a moderate risk of bias. For the one study with a comparison group, the MINORS score was 15/24, which again represents a moderate risk of bias. Furthermore, our literature search only yielded seven articles, which demonstrates the lack of research to date. This small number and their moderate risk of bias impacts the strength of conclusions that can be drawn from this review.

## Conclusions

Rectus plication is commonly performed during an abdominoplasty to improve form and function. While the literature to date is encouraging with respect to functional outcomes, improvements in strength outcomes are less consistent. Substantial between-study heterogeneity in patient population, outcome measures and control group limit the strength of our conclusions. Future research should involve a large, three-armed trial comparing abdominoplasty with rectus plication, abdominoplasty without rectus plication, and non-operative management (physiotherapy). Outcomes should include both objective and subjective strength outcomes, as well as patient-reported functional outcomes.

## Appendices

Appendix A. Protocol

Functional Improvement in Abdominal Strength Following Rectus Diastasis Repair for Abdominoplasty: A Systematic Review Protocol (OSF)

Study Information

Hypotheses: Patients who have undergone abdominoplasty or panniculectomy with repair of the rectus diastasis will have a significant improvement in their abdominal strength.

Design Plan

Study type: Meta-Analysis - A systematic review of published studies. Blinding: No blinding is involved in this study. Is there any additional blinding in this study? Systematic screening of the literature will be completed independently by two reviewers, who will be blinded to the other person's decisions.

Study Design

A systematic review of the literature looking at abdominal strength measures after rectus diastasis repair in patients who have undergone abdominoplasty, to determine if there is marked improvement.

Randomization: Not applicable.

Sampling Plan

Existing Data: Registration prior to analysis of the data Explanation of existing data: Not applicable.

Data Collection Procedures

Inclusion: Patients who are undergoing abdominoplasty or panniculectomy; Patients who had surgical repair of rectus abdominus diastasis; Studies that report outcome parameters relating to abdominal strength or functional measure of core strength; All time horizons. Exclusion: Case reports, opinion pieces, editorials and non-primary research (e.g., systematic reviews, scoping reviews, commentaries); Animal or pre-clinical studies; Studies in languages other than English; Overlapping reports of the same study. Data Collection: A study-specific data extraction form will be developed using Google Sheets (Google, California, USA). The form will be created a priori and any required modifications will be made by the reviewers in the extraction phase. The form will include study characteristics (design, date, location, sample size, demographics and level of evidence), specifics of abdominoplasty procedure, and reported study outcomes (function, abdominal strength, pain, disability, complications, etc.). The reported outcomes will be specific to each of the studies included in our review. Reviewers will independently collect data from each of the studies included using the form. Sample size: This is dependent on the number of studies yielded through our search. Sample size rationale: Not applicable. Stopping rule: Not applicable.

Variables

Manipulated variables: Not applicable. Measured variables: Primary Outcomes: Functional abdominal strength Objective (ex. Biodex system-4, trunk endurance) Subjective Secondary Outcomes: Subjective reports from patient on quality of life relating to core strength (ex. VAS, VHPQ, disability rating index, SF-36, EQ-5D) Complications (ex. Recurrence, SSI, seroma, hematoma, dehiscence, etc.).

Analysis Plan

Statistical models: We will summarize study characteristics using frequencies and percentages for categorical data and means and standard deviation or median and interquartile range for continuous data. We will summarize populations, interventions, comparison groups (if applicable) and reported outcomes in a table and we will present authors’ conclusions about the intervention graphically if relevant.

Transformations: No response

Inference criteria: No response

Data exclusion: No response

Missing data: We will contact the authors of the study for any missing data.

Exploratory analysis: No response

Level of evidence: Level of evidence will be classified as follows: Level I (randomized controlled trials/systematic reviews/meta-analysis of RCTs), Level II (cohort study), Level III (case control study), Level IV (case series/case report), or Level V (opinion/survey/qualitative study). Risk of bias in individual studies: Risk of bias for randomized control trials will be assessed using the Cochrane Risk of Bias tool. For non-randomized trials, the Methodological Index for Non-Randomized Studies (MINORS) will be used to assess risk of bias. Risk of bias across studies: The GRADE criteria will be used to consider risk of bias, inconsistency, indirectness, imprecision and likelihood of publication bias. This will be used to assess the quality of evidence, and taken into consideration for clinical recommendations.

Other

Contributors: Jessica Gormley

Registration type: OSF Preregistration

Date registered: April 29, 2020

Date created: April 29, 2020

Registered from: osf.io/gtw7j

Category: Project

Registration DOI: 10.17605/OSF.IO/H9JB3

Publication DOI: No publication DOI

Subjects: Medicine and Health Sciences

Affiliated institutions: This registration has no affiliated institutions

License: No license

Tags: No tags

Appendix B. Search Strategy

**Table 6:** Search Strategy Medline

Search Strategy
1. abdominoplasty/ or lipoabdominoplasty/
2. (abdominoplast* or panniculectom* or lipoabdominoplast*).mp.
3. Lipectomy/
4. (lipectom* or tummy-tuck* or body-contour* or abdominal-contour* or abdominal skin reduc*).mp.
5. (repair* or correct* or closure* or plicat* or restor* or mesh*).mp.
6. surgical mesh/
7. sutures/
8. suture techniques/
9. ((surg* or operat*) adj2 (management or correct*)).mp.
10. or/1-9
11. Diastasis, Muscle/
12. (muscle diastas?s or myodiastas?s).mp.
13. Rectus Abdominis/
14. ((recti or rectus or abdominis) adj4 (divarication* or diastas?s or divorce or separation or fascia or plicat*)).mp.
15. or/11-14
16. 10 and 15
17. rectus abdominis/su
18. 16 or 17
19. "Activities of Daily Living"/
20. activities of daily living.mp.
21. daily living activities.mp.
22. physical endurance/ or exercise tolerance/
23. (physical endurance or exercise tolerance).mp.
24. Disability Evaluation/
25. (disability adj2 (index* or evaluation*)).mp.
26. Self Report/
27. self-report*.mp.
28. Muscle Strength/
29. ((muscle or core or abdominal or trunk) adj2 (function or strength)).mp.
30. "Quality of Life"/
31. quality of life.mp.
32. or/19-31
33. 18 and 32
